# Protocol for comprehensive RNA sequencing analysis of murine long non-coding RNAs during aging

**DOI:** 10.1016/j.xpro.2021.100397

**Published:** 2021-03-20

**Authors:** Xinyue Lu, Qiuzhong Zhou, Jin Liu, Lei Sun

**Affiliations:** 1 School of Medicine, Shanghai Jiao Tong University, Shanghai 200025, China; 2 Cardiovascular and Metabolic Disorders Program, Duke-NUS Medical School, Singapore 169857, Singapore; 3 Centre for Quantitative Medicine, Health Services & Systems Research, Duke-NUS Medical School, Singapore 169857, Singapore

**Keywords:** Genomics, Sequencing, RNAseq

## Abstract

Comprehensive analyses of lncRNAs in aging have been lacking because previous studies have mainly focused on the protein-coding genes during aging. Here, we describe a protocol for the organism-wide analysis of murine lncRNAs during aging. We provide step-by-step instructions to identify lncRNAs that contribute to aging and to determine their underlying functions in each tissue. We further describe methods to compare the lncRNA expression patterns and dynamic changes among multiple tissues.

For complete details on the use and execution of this protocol, please refer to Zhou et al. (2020).

## Highlights

• Differential gene expression and aging-regulated lncRNA identification
• Functional annotation specific for lncRNAs
• Tissue-specific analysis for aging-regulated lncRNA

## Graphical abstract

**Figure:**
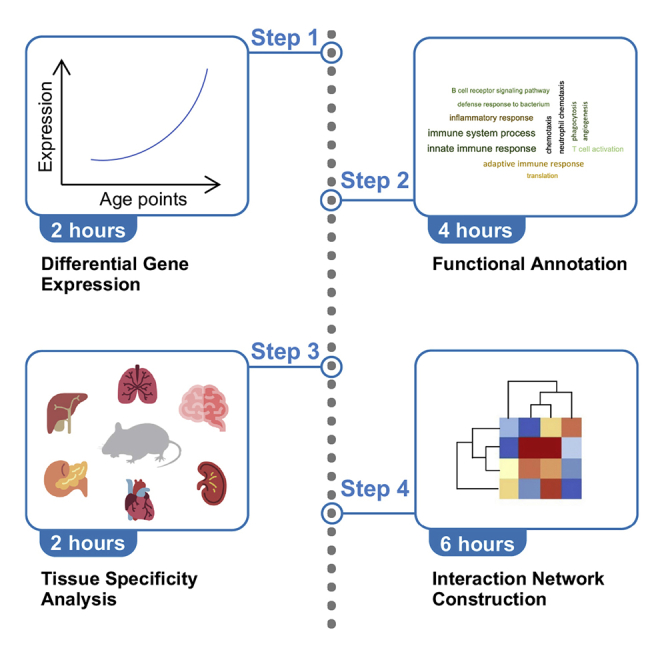

## Before you begin

### Overview of the project

**Figure 1:**
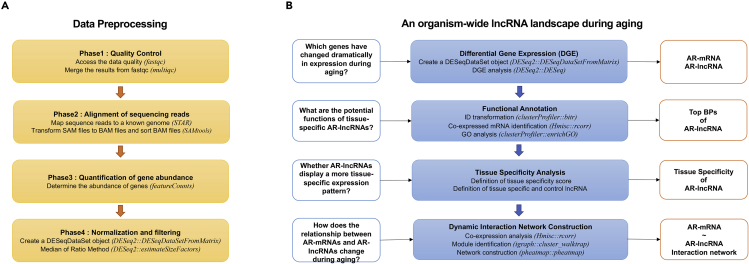
Overview of the project (A)A flowchart of data preprocessing. (B)A flowchart of data analysis of this lncRNA study.

This project aims to study the dynamic regulation of lncRNAs and their functional implications across multiple rodent tissues during aging. This study includes data collection, preprocessing (Figure 1A) and data analysis composed of differential gene expression analysis, functional annotation, tissue specificity analysis and dynamic network construction (Figure 1B).

For differential gene analysis, we make use of negative binomial model in DESeq package to identify AR(aging-regulated) mRNAs/lncRNAs in multiple tissues.

For functional annotation, we first use Pearson correlation to identify co-expressed mRNAs of each AR-mRNAs. Then we perform BP enrichment analysis to discover the role of those co-expressed mRNAs during aging, so as to infer the potential functions of each AR-lncRNA.

For tissue specificity analysis, we define and calculate the tissue specificity score of each lncRNA, which is used to compare the tissue specificity between AR-lncRNAs and ANR-lncRNAs/AR-mRNAs.

For dynamic network analysis, we first use Pearson correlation to calculate the relationship between each AR-mRNA and each AR-lncRNA at each age point and then make use of the Walktrap community finding algorithm in igraph package to identify modules where AR-mRNAs and AR-lncRNAs are connected.

### Data collection

In this protocol, we make use of RNA sequencing expression data to identify those lncRNAs functioning in aging among different tissues. Since correlation between lncRNAs and mRNAs is used to predict the regulatory roles of interested lncRNAs, the samples of interest should include RNA sequencing reads of both mRNAs and lncRNAs from a series of tissues in a specific species. In order to study the transcriptome alternations across the lifespan of a selected species, the samples should come from different age points for each tissue.

***Note:*** For example, we chose mouse as our focused species and considered 11 tissues in our published study. From 8, 26, 60, 78, 104 weeks, 5 replicates of each tissue were collected. That is, we had 5(replicates) × 5(different time points) × 11(different tissues)=275 samples in total. The number of lncRNAs was 21,981 and the number of mRNAs was 12,533, which were consistent in all samples.

***Note:*** The background among replicates should be matched as closely as possible.

### Data preprocessing

**Timing: 3-4 days**

***Note:*** Use conda create command in conda to create an environment specific for RNA-seq preprocessing.

***Note:*** We begin with FASTQ files. If the raw files are in the form of SRA, fasterq-dump in sratoolkit can be used to transform data formats.

1. Quality control
  a. Access the quality of data with fastqc (v0.11.9), which will generate two result files for each sample in the form of zip and html (Andrews, 2010).
  b. Merge the results from fastqc with multiqc (v1.9), which will result in a single quality control report across all samples in the form of html (Ewels et al., 2016).
    i. The report consists a series of items, showing data quality in multiple aspects. Mainly focus on Basic Statistics, Per Base Sequence Quality and Adapter Content.
    ii. If some of the samples failed to pass the above mentioned quality control items, using trim galore (v0.6.5) to apply adapter and quality trimming to raw fastq files (Krueger, 2012).
  c. Make sure all samples are of good quality by fastqc again.

***Note:*** Step-c requires repeating step-a and step-b(i). Only samples passing the mentioned items after adapter and quality trimming are considered as “of good quality” and used for following analysis.

2. Alignment of sequencing reads
  a. Use STAR (v2.7) to map sequence reads to a known genome according to the interested species (Dobin et al., 2013). The reference genome sequence can be downloaded from GENCODE (https://www.gencodegenes.org) (Frankish et al., 2019).
    ***Note:*** For example, we perform alignment by mouse reference genome (Release M17 of GenCode, GRCm38) with parameter of sjdbOverhang149 in our published study.
  b. Use SAMtools (v1.11) to deal with file format (Li et al., 2009).
    i. Transform SAM files, the output files of STAR, to BAM files by view command.
    ii. Sort BAM files by sort command.
      ***Note:*** BAM files are binary version of SAM files, which can increase the execution speed in gene abundance quantification.

3. Quantification of gene abundance
  a. Assign reads to genes and determine the abundance by featureCounts (v2.0.1) (Liao et al., 2014).
    i. Specific parameter -p counts fragments instead of reads. It is only applicable for pair-end reads since single-end reads will always be counted as reads.
    ii. Parameter -a should be followed by the name of the annotation file. (GTF file obtained in step2-a)
    iii. Parameter -o should be followed by the name of output file including read counts (txt format). Another file including summary statistics of counting results will also be generated.
  b. Use cut command to extract gene names and gene counts, from the output file (txt format) of featureCounts.

***Note:*** The 2^nd^ to 6^th^ columns include information of genes. Only gene names (the 1^st^ column) and gene counts of each sample (the 7^th^ to (7 +(*N*– 1 ))^th^ column, where *N* represents the total number of samples) is necessary.

***Note:*** This section will generate a *G* × *N* raw count matrix including samples from all of the tissues, containing the expression of mRNAs and lncRNAs, where *G* is the total number of mRNAs and lncRNAs and *N* is the total number of samples. To be specific, if there are *M* tissues and $N_{i}\left(i=1,2,\ldots ,M\right)$ samples for each of the tissues, $N=\sum N_{i}$.

**CRITICAL:** All of the following analysis will be executed in R statistical environment (v3.6.1).

***Note:*** In the following parts, we give variable names to some R objects , shown in the form of italics, to make our protocol more understandable. The names of R objects can be adjusted according to the projects.

***Note:*** In the following parts, R functions are in the form of ‘<functionname> command’.

4. Normalization and filtering troubleshooting 1 and 2
  a. Import raw read counts matrix as a dataframe *raw.*
  b. Perform normalization of raw read counts by the method of Median of Ratio in DESeq2 package(Anders and Huber, 2010).
    i. Use DESeqDataSetFromMatrix function to create a DESeqDataSet object *dds_norm.* The only necessary input is the dataframe *raw.*
    ii. Use estimateSizeFactors function with *dds_norm* to perform normalization. Then use counts function with a parameter of normalized=TRUE to obtain a normalized matrix *norm.*
  c. Carry out log transformation of matrix *norm* by the log1p function and only keep genes with expression > 0 in at least 20% samples for each tissue.
  d. Take the mean expression value for all the replicates at a specific time point for each tissue separately.

***Note:*** This section will generate a series of $g_{i}\times n_{i}\left(i=1,2,\ldots ,M\right)$ normalized count matrix $T_{i}\left(i=1,2,\ldots ,M\right)$for each of the tissues containing the expression of mRNA and lncRNA, where *M* is the total number of tissues, *g*_*i*_ is the number of filtered genes and *n*_*i*_ is the number of samples in this tissue. *n*_*i*_ is the number of different age points after taking the mean value of all the replicates.

***Note:*** It is recommended to use the list structure to store raw and processed counts (before and after taking the mean value) for each tissue. Intermediate results in subsequent parts can also be saved in this way.

## Key resources table

**Table:** 

REAGENT or RESOURCE	SOURCE	IDENTIFIER
**Deposited data**		

RNA-seq raw data are available at NGDC (http://bigd.big.ac.cn/) under the BioProject accession number PRJCA002140	Zhou et al., 2020	NGDC:PRJCA002140

**Software and algorithms**		

Quality control of RNA-seq : fastqc v0.11.9	Andrews, 2010	http://www.bioinformatics.babraham.ac.uk/projects/fastqc/
Summarize the output of fastqc : multiqc v1.9	Ewels et al., 2016	https://multiqc.info
Quality and adapter trimming to fastq files : Trim Galore v0.6.5	Krueger, 2012	http://www.bioinformatics.babraham.ac.uk/projects/trim_galore/
RNA-seq mapping : STAR v2.7	Dobin et al., 2013	https://github.com/alexdobin/STAR
Format transformation and sorting : SAMtools v1.11	Li et al., 2009	http://samtools.sourceforge.net
Counting reads : featureCounts v2.0.1	Liao et al., 2014	http://subread.sourceforge.net/
R system : R v4.0.2	N/A	https://cran.r-project.org/
Programming environment of R : RStudio v1.3.1093	N/A	https://rstudio.com/
Differential gene expression analysis : DESeq2 v1.28.1	Anders and Huber, 2010	https://bioconductor.org/packages/release/bioc/html/DESeq2.html
Functional annotation : clusterProfiler v3.16.1	Yu et al., 2012	https://bioconductor.org/packages/release/bioc/html/clusterProfiler.html
Gene expression interaction : Hmisc v4.4	Harrell, 2019	https://cran.r-project.org/web/packages/Hmisc
Network cluster : igraph v1.2.4.2	Csardi and Nepusz, 2006	https://igraph.org/redirect.html
Data visualization: pheatmap v1.0.12	Kolde, 2019	https://github.com/raivokolde/pheatmap

## Step-by-step method details

### Differential gene expression

**Timing: 2h**

***Note:*** Differential expression analysis is performed **in each tissue**.

***Note:*** Raw counts are used in this part.

**Figure 2:**
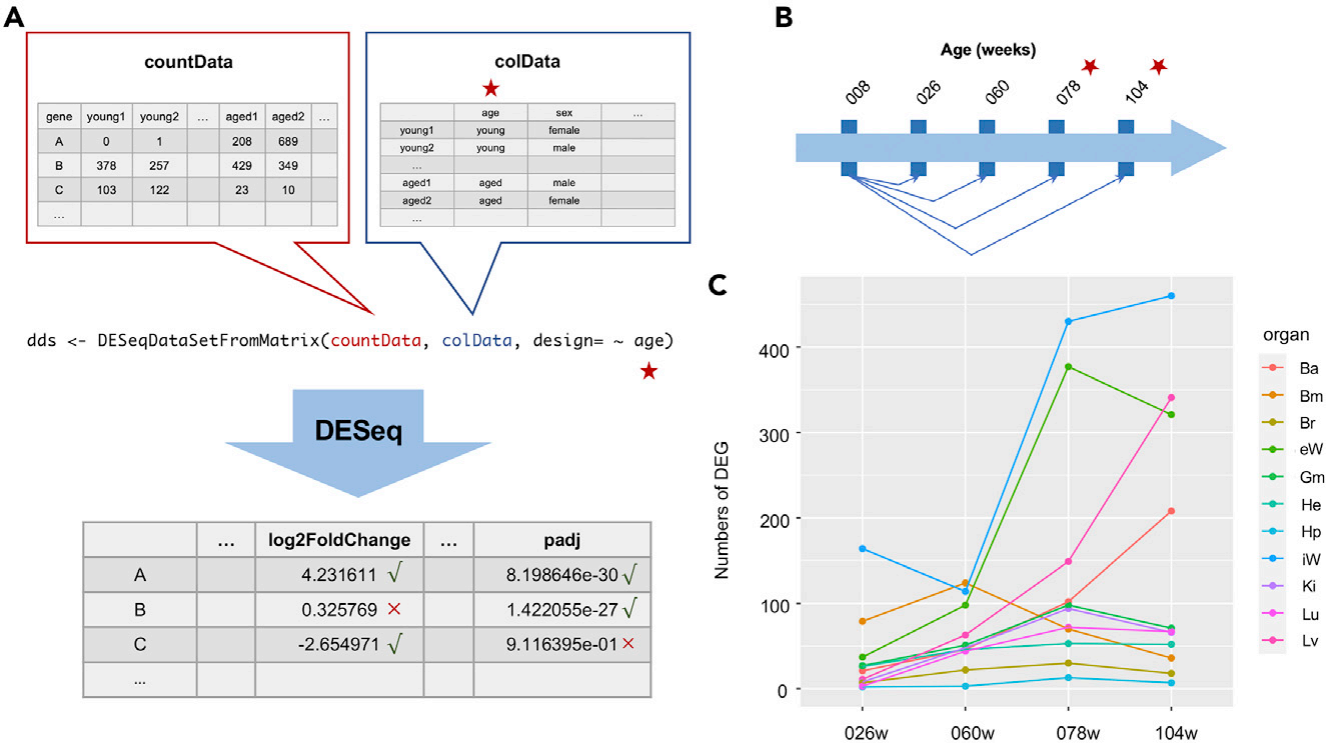
Detection of differentially expressed genes among multiple tissues (A)A graphical instruction of DESeq2 package. Here is an example of countData and colData, serving as essential input of constructing a DESeqDataSet object *dds*. For colData, we are only interested in the effect of aging here, as starred in the figure, so we just input age for the formula of design. DESeq function performs intermediate calculations. As seen in the sample outcomes, only gene A will be identified as DEG since it fulfills the criterion of |log2FC| ≥ 0.75 and FDR ≤ 0.05, but not gene B(|log2FC|<0.75) and gene C(adjusted p-value>0.05). (B)Illustration of pairwise comparison for differential gene expression. DEGs in 78-week samples and 104-week samples are identified as AR-genes, as starred in the figure. (C)Line plot demonstrating the differentially expressed lncRNA numbers of pairwise comparison in multiple tissues.

1. Perform pairwise differential gene expression of the youngest group with each aged group using DESeq2 (v1.28.1) package (Anders and Huber, 2010). (Figure 2A)
  a. Use DESeqDataSetFromMatrix function to create a DESeqDataSet object *dds.* The input arguments include *CountData*, *ColData* and *Design.*
    i. *CountData* is a matrix containing the raw read counts of selected groups. Filter the read counts to keep the same genes as the processed counts after data preprocessing. Each row represents a gene. Each column represents a sample in the interested young and aged groups.
    ii. *ColData* is a matrix containing sample information. Each row represents a sample, in the order of columns of countData. Each column represents a type of group information, including age and other covariates such as sex.
    iii. *Design* is a formula representing how the gene counts rely on the variables in colData. Include the interested variables or their interactions in this formula.
      ***Note:*** For example, to examine which genes are differentially expressed during aging, only need to use ∼age as the *design* (Figure 2A). If other covariates such as sex and sex:age interactions are considered, ∼age + sex + age:sex(as their interaction) should be used for the formula of *design*.
      ***Note:*** A DESeqDataSet object is used to store the input raw counts and intermediate results during the analysis of differential gene expression. All operations are performed on the DESeqDataSet object.
  b. Using DESeq function with *dds* to conduct the analysis of differential gene expression. Outcomes tables can be generated by results function. The tables contain log2FoldChange and adjusted p-value(FDR) of each gene.
    ***Note:*** DESeq function contains three steps for the analysis of differential gene expression (estimateSizeFactors function, estimateDispersions function, and nbinomWaldTest function). As a substitute, you can also execute the three functions step-by-step.
  c. For each of the aged group, the differentially expressed genes are identified with the criteria of |log_2_FC| ≥ 0.75 and FDR ≤ 0.05.

2. Differentially expressed genes in the interested aged groups are identified as aging-regulated (AR) genes.

***Note:*** Up/Down-regulated AR-genes can be identified according to the positive/negative log2FC values.

***Note:*** ln most organs, the number of differentially expressed lncRNAs increases during aging (Figure 2C). The differential expressed mRNA/lncRNA of 78 weeks or 104 weeks compared with 8 weeks are classified as aging-regulated mRNAs (**AR-mRNAs**)/Aging-regulated lncRNAs (**AR-lncRNAs**) (Figure 2B).

### Functional annotation

**Timing: 4h**

***Note:*** Functional annotation is performed **in each tissue**.

***Note:*** The mean value of processed counts of different replicates are used in this part.

***Note:*** The clusterProfiler (v3.16.1) package requires the gene ID to be EntrezID for the functional annotation. If the type of gene ID is already EntrezID, please skip step 3.

**Figure 3:**
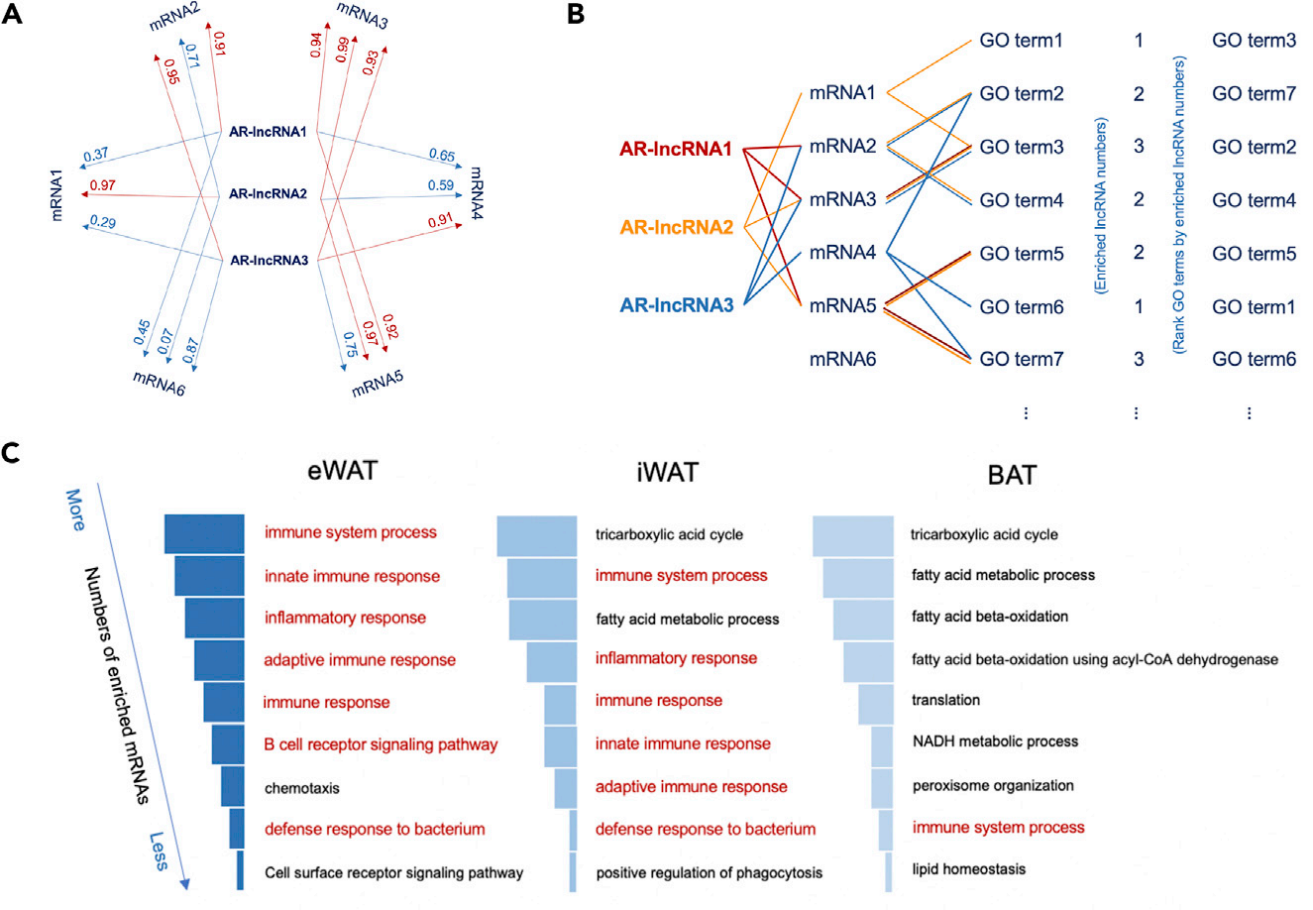
Functional annotation of aging-regulated lncRNAs (A) A graphical instruction of co-expressed mRNAs identification. Here is an example considering 3 AR-lncRNAs and 6 mRNAs. Their |correlation coefficient|(FDR) are displayed correspondly in the figure. Only mRNAs with |correlation coefficient| ≥ 0.8 and FDR ≤ 0.05 with an AR-lncRNA are selected as co-expressed mRNAs of a specific AR-lncRNA, shown as red lines. To be specific, mRNA1 is a co-expressed mRNA of AR-lncRNA2, but not of AR-lncRNA1 and AR-lncRNA3. (B) A graphical instruction of BP enrichment for AR-lncRNAs. Firstly, obtain significantly enriched BP terms of co-expressed mRNAs for each AR-lncRNA. To be specific, GO term3 is both enriched in mRNA1 and mRNA3. mRNA1 is co-expressed with AR-lncRNA1, and mRNA3 is co-expressed with AR-lncRNA1,2,3. As a result, GO term3 is associated with all of the 3 AR-lncRNAs. Secondly, rank each BP term decreasingly according to the number of enriched AR-lncRNAs. (C) Comparison of top BPs in adipose tissues. Most of top BPs are associated with immune response.

3. Transform gene ID to EntrezID for mRNAs using the bitr function in the clusterProfiler package (Yu et al., 2012).
  a. Find genome-wide annotation package for the species of your experimental model from http://bioconductor.org/packages/release/BiocViews.html#___OrgDb.
    ***Note:*** A series of org.Xx.eg.db packages including annotation for common species are required when performing the transformation of gene ID type. For example, we chose org.Mm.eg.db as our published study was conducted on mice.
  b. Use bitr function to perform ID transformation. The parameters, including geneID, fromType, toType and OrgDb, are used for the essential inputs.
    i. Input your gene id for ‘geneID’.
    ii. Use keytypes function to see the supported ID types for ‘fromType’ and ‘toType’.
    iii. The name of selected org.Xx.eg.db package is used for ‘OrgDb’.
      ***Note:*** The following GO analysis is performed for each set of co-expressed mRNAs of each AR-lncRNA.
      ***Note:*** It is recommended to execute the following steps of functional annotation based on the loop structure, since it is a great deal of work to conduct functional annotation for co-expressed mRNAs of each AR-lncRNA separately and integrate the results.

4. Identify the co-expressed mRNAs of each AR-lncRNA (Necsulea et al., 2014) (Figure 3A).
  a. Perform Pearson correlation between AR-lncRNAs and mRNAs and calculate p-value using rcorr function in Hmisc (v4.4) package. ((Harrell, 2019); (Schaum et al., 2019)) Take the absolute value of correlation coefficient. Using p.adjust function in stat package to adjust p-value by the method ‘fdr’.
  b. For each AR-lncRNA, its co-expressed mRNAs were selected with a cutoff at absolute Pearson correlation coefficient ≥ 0.8 and FDR ≤ 0.05 in each tissue.

5. Perform GO analysis by clusterProfiler package (Yu et al., 2012).
  a. Use enrichGO function to perform BP(biological process) enrichment in GO analysis. Set ‘ont’ as ‘BP’ and ‘qvalueCutoff’ as 0.05. This step will return enrichment GO categories, *go.*
    ***Note:*** The parameter ‘ont’ can be selected as ‘MF’ for molecular function, ‘BP’ for biological process and ‘CC’ for cell component, or can be selected as ‘All’ to contain all of three. BP enrichment is most widely used.
  b. Use data.frame function to turn *go* into the form of data frame, *go1.* Filter *go1* to remain columns including ‘ID’, ‘Description’, ‘p.adjust’, ‘geneID’ and ‘Count’.

***Note:*** The *go1* data frame for each AR-IncRNA can be combined during each loop by rbind function, which faciliates the following steps.

6. Analysis of the number of lncRNAs associated with each BP terms. (Figure 3B)
  a. Calculate the total number of associated AR-lncRNAs for each of the significantly enriched BP terms.
  b. Rank the BP terms according to the decreasing order of associated AR-lncRNA numbers.

### Tissue specificity analysis

**Timing: 2h**

***Note:*** Tissue specificity analysis is performed **at each time point**.

***Note:*** The mean values of processed counts of replicates are used in this part.

7. Definition of tissue specificity score (Alvarez-Dominguez et al., 2015; Ding et al., 2018)

In the following equations, *n* represents the total number of tissues, *T*_*ij*_ represents the average expression of a given gene i in a given tissue j. (Figure 3A)

$$\mathrm{Tissue}\;\mathrm{fractio}n_{\mathrm{ij}}=\frac{T_{ij}}{\sum_{j=1}^{n}T_{ij}}$$

$$\mathrm{Tissue}\;\mathrm{specificity}\;\mathrm{scor}e_{i}=\max\left\{\mathrm{Tissue}\;\mathrm{fractio}n_{\mathrm{ij}}\right\}$$

***Note:*** The highest tissue fraction of a lncRNA, which indicates that a given lncRNA is highest expressed in a given tissue, is used as its tissue specific score. It can be used to compare the expression of interested lncRNAs among different tissues.

8. Definition of tissue specific and control lncRNA
  a. Rank all lncRNAs according to tissue specificity scores in a decreasing order.
    ***Note:*** Use order function with parameter ‘decreasing = T’ to sort your data decreasingly.
  b. Define the top 20% lncRNAs as tissue specific lncRNAs and the bottom 20% as control lncRNAs.

### Dynamic interaction network construction

**Timing: 6h**

***Note:*** Interaction network between AR-lncRNAs and AR-mRNAs is constructed **in each tissue**.

***Note:*** Processed counts without taking the mean value of replicates are used in this part.

**Figure 5:**
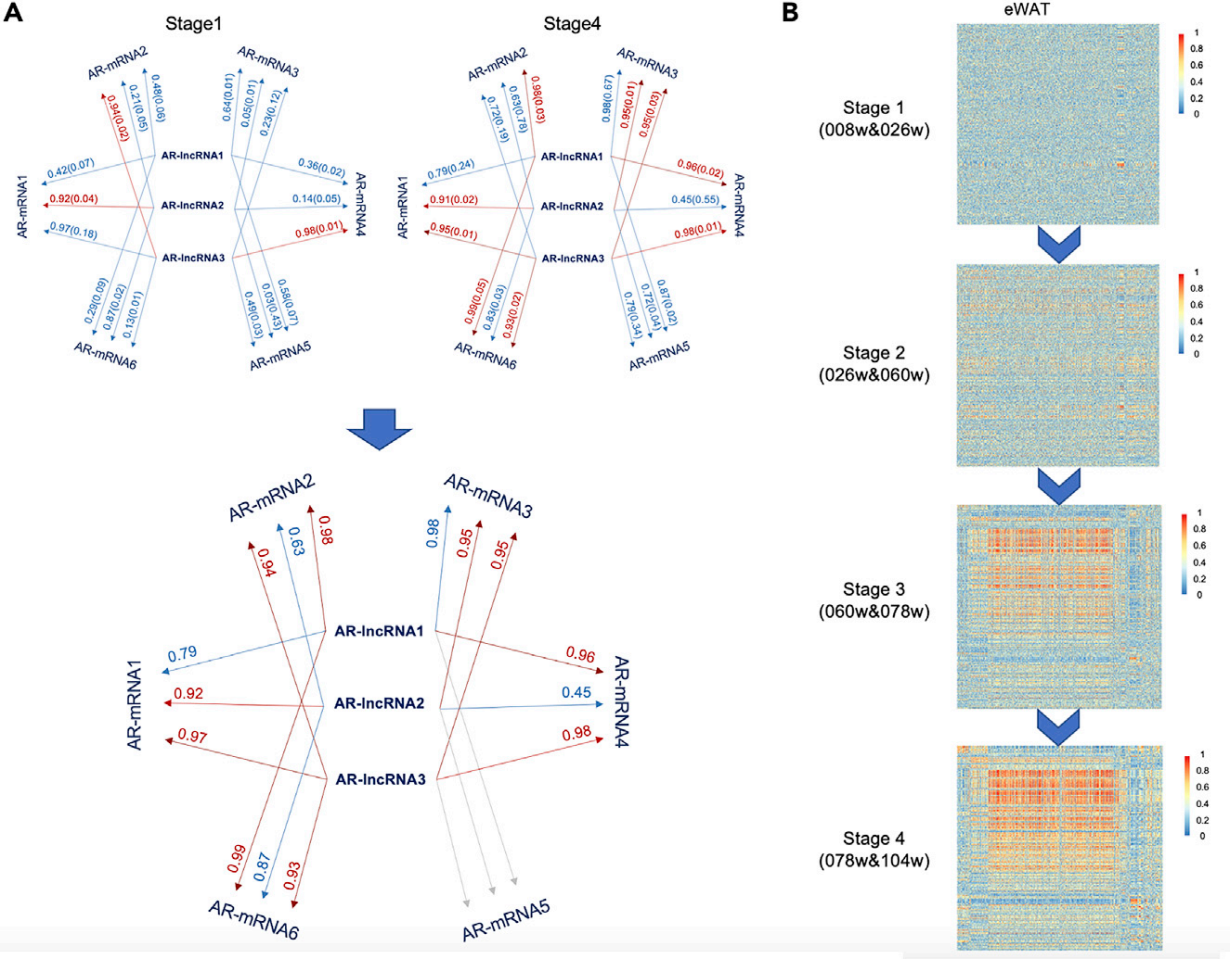
Dynamic AR-lncRNA~AR-mRNA crosstalk during aging (A) A graphical instruction of AR-genes filtering for module detection. Here is an example considering 3 AR-lncRNAs and 6 AR-mRNAs. Their |correlation coefficient|(FDR) are displayed correspondly in the figure. Firstly, pick up edges between an AR-lncRNA and an AR-mRNA with |correlation coefficient| ≥ 0.9 and FDR ≤ 0.05 in Stage1 and Stage4, separately, shown as red lines. To be specific, the edge between AR-lncRNA2 and AR-mRNA1 is taken since it fulfills both criterions. On the contrary, though AR-lncRNA3 and AR-mRNA1 has a |correlation coefficient| = 0.97 > 0.9, the edge is not selected since its FDR = 0.18 > 0.05. Secondly, if an edge is chosen in either or both of Stage1 or Stage4, include it for consequent steps. It can be clearly seen that AR-mRNA5 is not correlated with any AR-lncRNA. As a result, remove AR-mRNA5 from network construction. For the remaining edges, use the higher value of |correlation coefficient|of Stage1 and Stage4 for module detection. Take the edge between AR-lncRNA2 and AR-mRNA1 as an example. It is picked both in Stage1 and Stage4 and remains in the network. Its |correlation coefficient| in stage1(0.92) is higher than stage4(0.91), keep 0.92 rather than 0.91. (B) Dynamic network showing the interaction of AR-lncRNAs and AR-mRNAs during the mouse lifespan in eWAT. Each row represents an AR-lncRNA. Each column represents an AR-mRNA.
    ii. Use *graph_from_data_frame* function to create an igraph graph *g* with the dataframe *exp.*
    iii. Use cluster_walktrap function to identify modules of *g* with the parameter of steps=10, which will return a community object *wc.* Use membership function to obtain the module information for *wc.* Use a new dataframe *mod* to store these information.

***Note:*** The step highly depends on computing resources.

***Note:*** The cluster_walktrap function only keeps modules with gene number ≥ 30.

9. Identification of modules in dynamic interaction networks
  a. Calculate the Z-score for the log-transformed normalized counts of each AR-lncRNA and AR-mRNA at different age points, separately. In the following equation, *μ* represents the mean value, σ represents the standard deviation.
    $$Z-score=\frac{x-\mu }{\sigma }$$
    ***Note:*** Processed data points after Z-score normalization follows the standard normal distribution. Here, Z-score normalization enables us to compare expression data at different age points on the same level.
  b. Combine the samples from the neighbor age points into 4 stages (stage1: 008w&026w, stage2: 026w&060w, stage3: 060w&078w, and stage4: 078w&104w).
    ***Note:*** We performed the analysis for the combined samples as our sample size was relatively small (n=5) and may not provide accurate outcomes. If you have ≥ 10 samples for each age points, you can skip this step.
  c. For expression matrix in each stage, evaluate Pearson correlation for each AR-lncRNA and AR-mRNA using rcorr function in Hmisc package ((Harrell, 2019); (Schaum et al., 2019)). Take the absolute value of correlation coefficient and obtain four correlation matrixes *cor1*, *cor2*, *cor3*, *cor4.* Use p.adjust function in stat package to adjust p-value by the method ‘fdr’.
  d. When a pair of AR-lncRNA and AR-mRNA has the absolute correlation coefficient ≥ 0.9 and adjusted p-value ≤ 0.05 in either or both of stage1 or stage4, keep the AR-lncRNA and AR-mRNA pair in the subsequent dynamic network construction.
  e. Identify modules for dynamic network construction using igraph (v1.2.4.2) package (Csardi and Nepusz, 2006).
    i. Construct a new dataframe *exp* to store higher correlation coefficient in stage1 and stage4 between each pair of filtered AR-lncRNA and AR-mRNA, where each line of the first and second column represents pairs of AR-lncRNA and AR-mRNA, the third column represents their correlation coefficient. (Figure 5A)

10. Construction of the AR-lncRNA∼AR-mRNA network
  a. Rearrange the rows and columns of correlation coefficient matrix in each stage, which adjusts the order of AR-lncRNAs and AR-mRNAs, in the order of module information (from module 1 to module n) of each AR-lncRNAs and AR-mRNAs in dataframe *mod.*
    ***Note:*** The order of AR-lncRNAs and AR-mRNAs become exactly the same in four different stages.
  b. Use pheatmap function in pheatmap package (v1.0.12) to plot heatmap of *cor1*, *cor2*, *cor3*, *cor4.* Set cluster_rows = F,cluster_cols = F.

## Expected outcomes

The differential gene expression analysis will lead to a list of differential expressed mRNAs/lncRNAs at different age points in each tissue (Figure 2C). The differentially expressed mRNAs/lncRNAs in your interested aged groups, identified as AR-mRNAs/AR-lncRNAs, are critical for downstream analysis.

The functional annotation identifies the BP terms that are associated with AR-lncRNAs. You can also make comparisons of top BPs connected with AR-lncRNAs between different tissues (Figure 3C).

**Figure 4:**
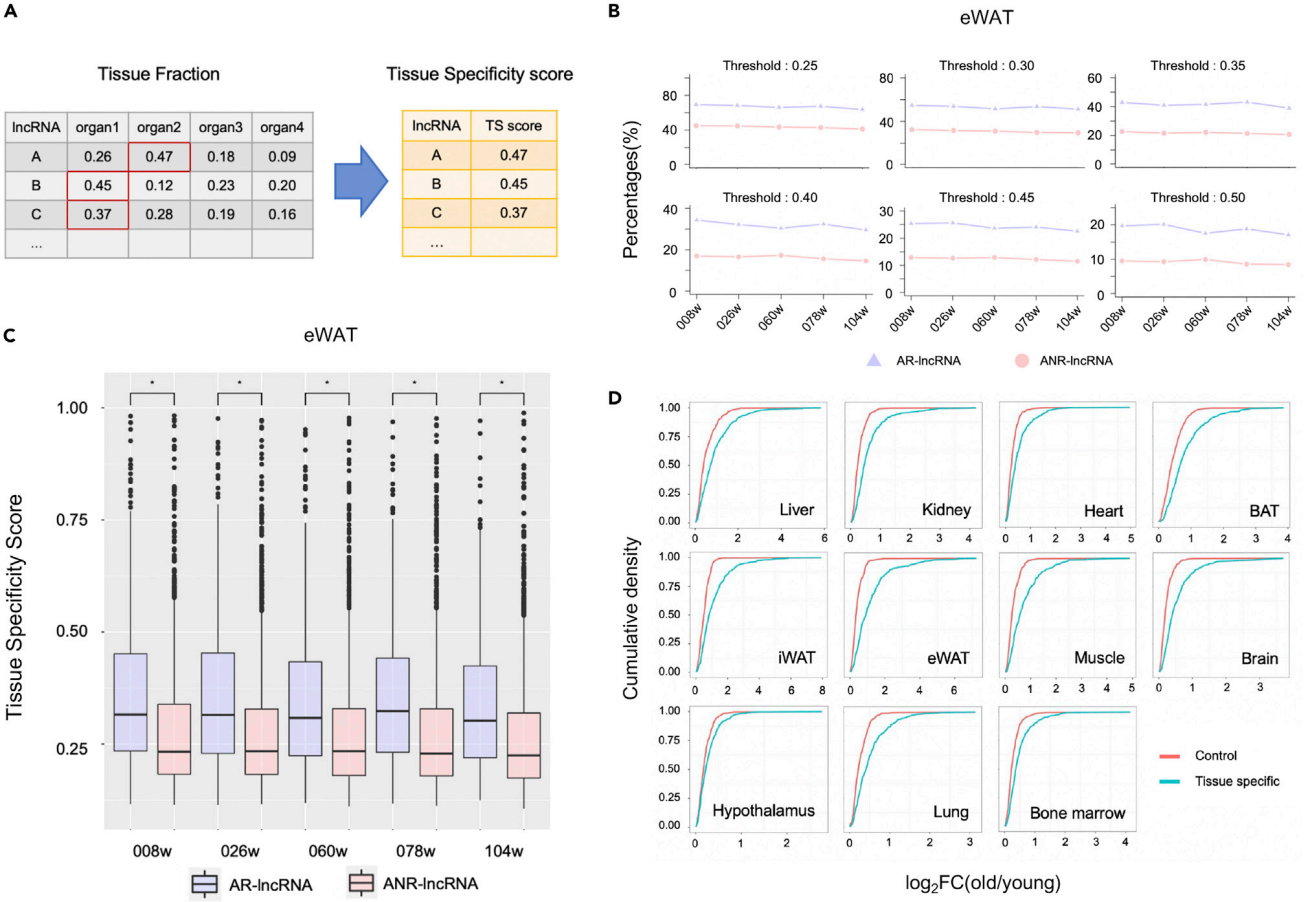
Tissue specificity analysis of lncRNA expression (A) A graphical instruction of tissue specific score calculation. Here is an example of tissue fraction matrix. The maximum tissue fraction for each lncRNA is selected for its tissue specificity score. (B) Proportion of tissue-specific lncRNAs in AR-lncRNAs and ANR-lncRNAs using different thresholds of tissue-specific score(0.25, 0.30, 0.35, 0.40, 0.45, 0.50) in eWAT. To be specific, if you choose 0.25 as the threshold, all lncRNAs with a tissue specific score ≥ 0.25 will be identified as tissue-specific lncRNAs, while the others are tissue-nonspecific lncRNAs. (C) The comparison of tissue specific score in AR-lncRNAs and ANR-lncRNAs in eWAT. ∗ represents p < 0.05, using Mann-Whitney test. (D) Cumulative density of |log2(old/young)| expression value of tissue-specific and control lncRNAs among multiple tissues. |log2(old/young)| is selected as the maximum |log2FC| between 8-week samples and 78-week/104-week samples.

The tissue specificity analysis calculates the tissue specificity scores for each lncRNA at different age points. These score can be used to investigate the tissue specificity of AR-lncRNAs (Figures 4B and 4C). Comparision of aging-induced changes between tissue specific and non-specific lncRNAs indicates that tissue-specific lncRNAs tend to be more regulated during aging (Figure 4D).

The dynamic interaction network construction can reveal the modules of highly correlated AR-lncRNAs and AR-mRNAs for different tissues at different age stages. The growth of modules generally indicates the enhanced functional and regulatory relationship(Figure 5B). Functional annotation can be performed within drastically growing modules in each tissue to infer the functional changes during aging.

## Limitations

The quality of raw data have a strong influence on the analysis, e.g., level of abundance, batch effect, duplicate rate. A principal component analysis (PCA) can be performed by plotPCA function in DESeq2 package on normalized counts. If the data are of good quality, transcriptomes are likely to be grouped according to organ identity instead of age points.

## Troubleshooting

### Problem 1

Removing batch effect.

### Potential solution

Batch effect results from a subset of experiments running on different days, by different technicians or using different reagents, chips or instruments. Measurements with batch effect will have qualitatively different behaviours among conditions, which are uncorrelated with experimental variables in a particular study.

If the sample are not from the same batches, the removeBatchEffect function in limma package can help to solve this problem. Input the raw data and batch information and execute this function to remove batch effect, which can ensure the robustness of following analysis.

### Problem 2

Selection of normalization methods.

### Potential solution

Comparison of common normalization methods are listed below (Tables 1, 2, and 3).

To make the gene expression data more comparable within or between samples, normalization is often a prerequisite. The main factors that normalization methods accounted for include sequencing depth, gene length and RNA composition.

In practice of TMM method, which scaling to library size, the gene length is absorbed into the parameter of the number of transcripts. As a result, TMM method will not use gene length as an input (Robinson and Oshlack, 2010). However, longer genes will have more read counts compared to shorter ones at the same expression levels. Thus, intrasample comparison may not be accurate. We used log2(FPKM) with a prior count of 0.5 to perform further normalization after TMM in our published study.

In this protocol, we make a slight change here by using Median of ratios method in DESeq2 package for normalization. It was reported that among all common normalization methods, only Median of ratios and TMM performed well in both differential expressed genes detection and false positive rate control (Dillies et al., 2012). So this change will not reduce the power of following analysis.

## Resource availability

### Lead contact

Further information and requests for resources and reagents should be directed to and will be fulfilled by the lead contact, Dr.Lei Sun (sun.lei@duke-nus.edu.sg).

### Materials availability

This study did not generate new unique reagents.

### Data and code availability

An example RNA-seq raw data is NGDC: PRJCA002140. An example code for this protocol is on https://github.com/Xinyue-Lu.

**Table 1:** Description of common types of normalization methods

Method	R package	Details
CPM	edgeR	Counts per million
TPM	N/A	Counts per length of transcript per million reads mapped
RPKM/FPKM	edgeR	
Median of ratios	DESeq2	Counts divided by size factors determined by taking the median of the ratios of observed counts
TMM	edgeR	Scaling to library size by size factors determined by using a weighted trimmed mean of the log expression ratio between samples

**Table 2:** Accounted normalization factors of common types of normalization methods

Method	Sequencing depth	Gene length	RNA composition
CPM	√	×	×
TPM	√	√	×
RPKM/FPKM	√	√	×
Median of ratios	√	×	√
TMM	√	×	√

**Table 3:** Recommend usage of common types of normalization methods

Method	Within sample comparison	Between sample comparison	Differential expression analysis
CPM	×	√	×
TPM	√	√	×
RPKM/FPKM	√	×	×
Median of ratios	×	√	√
TMM	×	√	√
